# Comparability of HbA1c and lipids measured with dried blood spot versus venous samples: a systematic review and meta-analysis

**DOI:** 10.1186/1472-6890-14-21

**Published:** 2014-05-12

**Authors:** Eshan T Affan, Devarsetty Praveen, Clara K Chow, Bruce C Neal

**Affiliations:** 1 The George Institute for Global Health, Missenden Rd, PO Box M201, NSW 2050 Sydney, Australia; 2 The University of Sydney, Sydney, Australia; 3 Westmead Hospital, Sydney, Australia

**Keywords:** Dried blood spot, HbA1c, Lipids, Meta-analysis, Total cholesterol, Triglycerides

## Abstract

**Background:**

Levels of haemoglobin A1_c_ (HbA1_c_) and blood lipids are important determinants of risk in patients with diabetes. Standard analysis methods based upon venous blood samples can be logistically challenging in resource-poor settings where much of the diabetes epidemic is occurring. Dried blood spots (DBS) provide a simple alternative method for sample collection but the comparability of data from analyses based on DBS is not well established.

**Methods:**

We conducted a systematic review and meta-analysis to define the association of findings for HbA1_c_ and blood lipids for analyses based upon standard methods compared to DBS. The Cochrane, Embase and Medline databases were searched for relevant reports and summary regression lines were estimated.

**Results:**

705 abstracts were found by the initial electronic search with 6 further reports identified by manual review of the full papers. 16 studies provided data for one or more outcomes of interest. There was a close agreement between the results for HbA1_c_ assays based on venous and DBS samples (DBS = 0.9858venous + 0.3809), except for assays based upon affinity chromatography. Significant adjustment was required for assays of total cholesterol (DBS = 0.6807venous + 1.151) but results for triglycerides (DBS = 0.9557venous + 0.1427) were directly comparable.

**Conclusions:**

For HbA1_c_ and selected blood lipids, assays based on DBS samples are clearly associated with assays based on standard venous samples. There are, however, significant uncertainties about the nature of these associations and there is a need for standardisation of the sample collection, transportation, storage and analysis methods before the technique can be considered mainstream. This should be a research priority because better elucidation of metabolic risks in resource poor settings, where venous sampling is infeasible, will be key to addressing the global epidemic of cardiovascular diseases.

## Background

Cardiovascular diseases [1] are increasing particularly rapidly in developing country settings with diabetes a key determinant of risk [2]. Documenting the role of dysglycaemia and other metabolic risk factors [3-8] can be challenging in these countries because the infrastructure and resources required to conduct research are limited. For example, assays of glycosylated haemoglobin (HbA1_c_) and blood lipids are usually done on venous blood samples which can be difficult to collect, transport and store. The use of dried blood spot sampling (DBS) [9,10] is one possible solution. DBS involves pricking the participant’s finger with a lancet and collecting drops of blood on a piece of filter paper. Samples are then dried and placed in sealed plastic bags for transportation and storage [11,12]. Compared to venous samples, collecting DBS requires minimal training of staff, is cheaper, is safer, provides for simpler transportation and is more acceptable to study participants [12-15].

DBS samples are now widely used for measuring serum antibodies, human immunodeficiency virus (HIV) loads and blood hormone levels with good data to define the comparability of results between analyses based upon DBS and standard venous samples [11,14,16,17]. The absence of comparable data to define the associations for HbA1_c_ and blood lipids means that DBS samples are not widely used in studies making assessment of cardiovascular risks. The objective of this project was to synthesise the available evidence describing the comparability of findings for assays of HbA1_c_ and blood lipids based upon DBS samples compared to standard venous samples.

## Methods

This project was a systematic review and meta-analysis done to define the association of findings for HbA1_c_ and blood lipids for analyses based upon standard venous samples compared to DBS samples. This was a secondary analysis of existing published data and no ethics review was therefore required.

### Search strategy

The Cochrane, Embase and Medline databases were searched electronically during July 2012 using combinations of the terms “dried blood spot”, “dried blood”, “DBS”, “filter paper”, “triglycerides”, “triacylglycerides”, “HbA1c”, “glycosylated haemoglobin”, “glycosylated hemoglobin, “glycated haemoglobin”, “glycated hemoglobin, “cholesterol”, “high density lipoprotein”, “HDL”, “low density lipoprotein” or “LDL”. Additional studies were identified by a manual examination of the reference lists of all studies identified as eligible.

### Eligibility criteria

Studies were eligible for inclusion if they directly compared values generated from analyses based on DBS samples to analyses based on venous samples. To be included, a study had to report in the form of a regression equation an association for one or more of the specified outcomes. There was no restriction on the type of study population.

### Data extraction

Two independent observers (EA and PD) reviewed the abstracts for eligibility and extracted standardised data into a data collection sheet for eligible studies. The data sought from each study were based upon a comparable prior systematic review done in the HIV field [17] and included: date of publication, study size, participant characteristics and sample storage conditions. For each risk factor reported upon we sought to identify the laboratory extraction method, biochemical assay method and regression coefficient. Where available we also noted data describing the stability of the DBS samples.

### Outcomes

The outcomes studied in this overview were HbA1_c_, total cholesterol, high density lipoprotein (HDL) cholesterol, low density lipoprotein (LDL) cholesterol and triglycerides.

### Statistical analysis

The characteristics of included studies were summarised in tabular form (Table 1). The linear regression coefficients from each study were pooled separately for each risk factor using a weighted least squares approach [18] to estimate an overall coefficient. The same method was used to estimate a combined intercept. This gave a relationship of the form: DBS = *b*Venous + *a* where ‘*b*’ is the combined coefficient and ‘*a*’ is the combined intercept. The synthesis of the parameters was done as follows:

$$b=\frac{\sum w_{i}b_{i}}{\sum w_{i}}\;\mathrm{and}\;a=\frac{\sum w_{i}a_{i}}{\sum w_{i}}$$

where the weight, *w*_*i*_ = the number of participants in study i, and b_i_ and a_i_ represent the coefficient and intercept, respectively, for the regression line in study i.

**Table 1:** Characteristics and findings of included studies

	**N**	**Diabetes**	**Population source**	**Mean (SD) of DBS values**	**Regression equation**
**HbA1**_**c**_					
Anjali 2007 [19]	78	Yes	-	9.45 (±1.86)	DBS = 0.95 V + 1.4
Buxton 2009 [20]	115	-	Hospital	-	DBS = 0.85 V + 0.81
Egier 2011 [9]	85	-	-	-	DBS = 0.933 V + 0.4
Fokkema 2009 [21]*	93	-	Community	-	DBS = 1.006 V - 0.092
Fokkema 2009 [21]*	88	-	Community	-	DBS = 0.994 V + 0.057
Fokkema. 2009 [21]*	73	-	Community	-	DBS = 0.987 V - 0.011
Gay 1990 [22]	58	Yes	Community	10.8 (±2)	DBS = 0.8 V + 1.8
Jeppsson 1996 [23]	41	-	-	7.49	DBS = 0.99 V + 0.16
Jones 2010 [10]*	73	-	-	6.74	DBS = 0.984 V + 0.189
Jones 2010 [10]*	40	-	-	8	DBS = 0.998 V - 0.204
Lacher et al. 2013 [24]	386	-	Community	5.92 (±1.2)	DBS = 0.94 V + 0.37
Lakshmy 2009 [25]	30	-	Community	5.94 (±1.58)	DBS = 0.9886 V + 0.0018
Little 1986 [26]	78	Yes/No	-	10.2	DBS = 1.09 V + 2.17
Lomeo 2008 [27]	97	-	-	-	DBS = 0.877 V + 1.09
Tabatabaei-Malazy 2011 [28]*	33	Yes	Community	8.8 (±1.6)	DBS = 1.20 V - 0.635
Tabatabaei-Malazy 2011 [28]*	33	Yes	-	8.9 (±1.7)	DBS = 1.25 V - 1.09
Wikblad 1998 [29]	145	Yes	-	-	DBS = 1.03 V - 0.405
**Triglycerides**					
Lakshmy 2010 [30]*	85	-	Community	1.6 (±0.6)	DBS = 1.028 V - 0.1690
Lakshmy 2012 [31]*	613	-	Community	1.16 to 1.87 (±0.45 to 0.79)	DBS = 0.9549 V + 0.1875
Quraishi 2006 [32]	75	-	Community	1.297 (±0.53)	DBS = 0.88 V + 0.13
**Total Cholesterol**			-		
Lacher 2013 [24]	395	-	Community	3.76 (±0.87)	DBS = 0.52 V + 1.08
Lakshmy 2010 [30]*	85	-	Community	5 (±1)	DBS = 0.727 V + 1.170
Lakshmy 2012 [31]*	613	-	Community	-	DBS = 0.7779 V + 1.1943
**HDL**					
Lacher 2013 [24]	395		Community	1.41 (±0.42)	DBS = 0.7 V + 0.46

*Some studies provided multiple estimates and are repeated in the table. SD = standard deviation, DBS = dried blood spot, V = venous.

Heterogeneity of the individual study estimates contributing to each meta-analysis was assessed using the Cochran’s Q and I^2^ statistics. Subsidiary analyses were done to explore the impact of assay method for the outcome of HbA1_c_.

## Results

**Figure 1:**
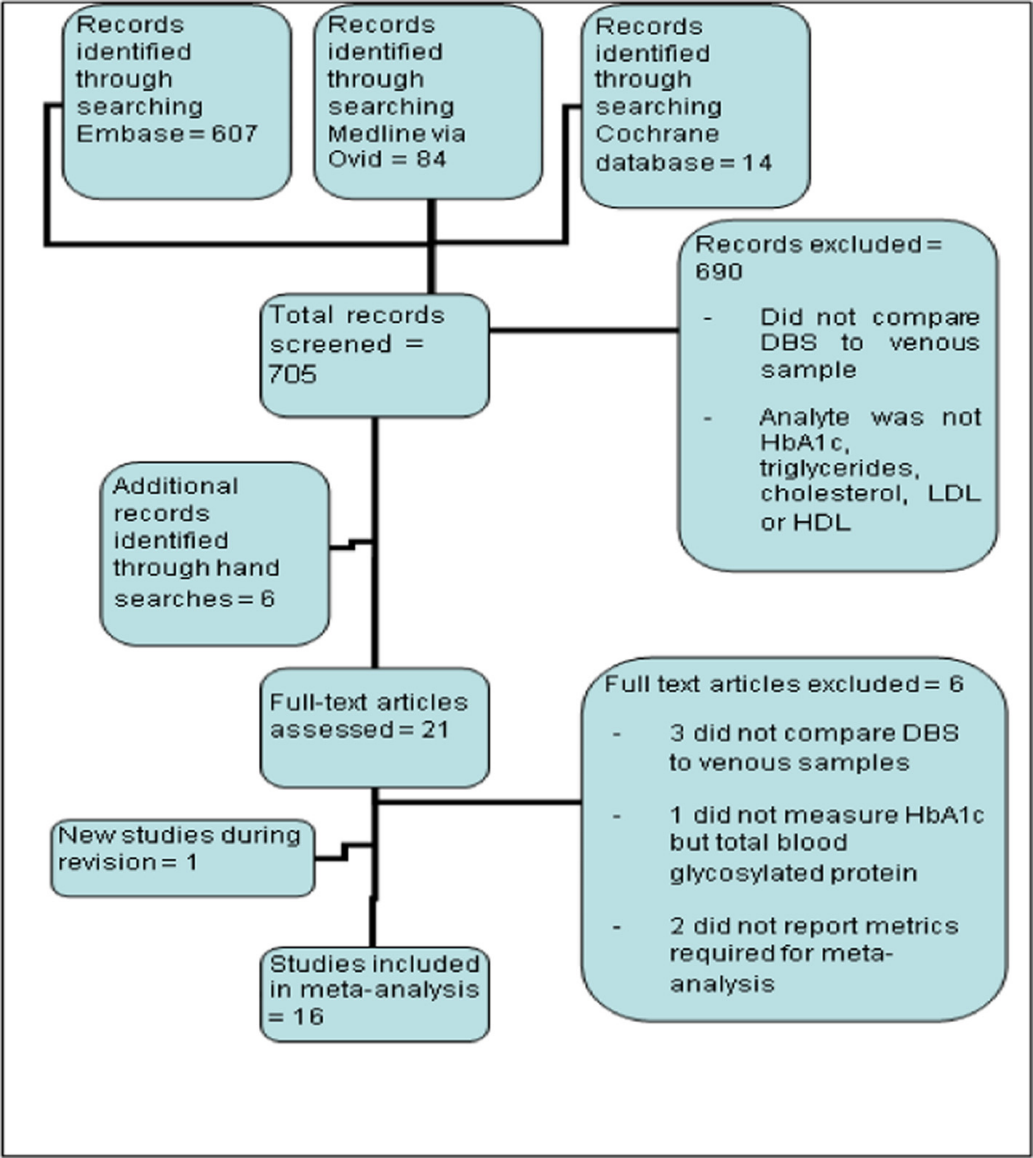
**Flow chart detailing identification of studies**[36]**.**

There were 705 records identified by the electronic search for which abstracts were reviewed. Six further studies were found by the manual search of reference lists for included studies (Figure 1). One final study was found during the review process. Sixteen studies were ultimately included in the meta-analysis, 12 of which reported necessary data for HbA1_c_, 1 for triglycerides, 2 for both triglycerides and total cholesterol and 1 for HbA1_c_, total cholesterol and HDL (Tables 1, 2) [9,10,19,21-34]. One other study of HbA1_c_ was excluded because it did not provide a regression equation [35] and one other study of triglycerides was excluded because it did not provide the sample size [34]. There were no studies reporting data for LDL-cholesterol identified.

**Table 2:** Methods of sample analysis in the different studies AC = affinity chromatography, TII = turbidimetric inhibition immunoassay, Ion = ion exchange chromatography, HPLC = high performance liquid chromatography, HR = haemolysing reagent, Col = colorimetry

	**Dried blood spot data collection details**			**Assay methods**	
	**Drying**	**Transportation**	**Extraction method**	**DBS**	**Venous**
**HbA1**_**c**_					
Anjali 2007 [19]	-	-	Anti-HbA1c antibody.	TII	TII
Buxton 2009 [20]	-	Special shipping	Roche HbA1c HR.	TII	HPLC
Egier 2011 [9]	-	-	Extraction buffer	HPLC	HPLC
Fokkema 2009 [21]*	-	-	HR	TII	TII
Fokkema 2009 [21]*	-	Routine mail	HR	TII	TII
Fokkema. 2009 [21]*	-	-		TII	TII
Gay 1990 [22]	Yes	Routine mail	-	AC	Ion
Jeppsson 1996 [23]	-	Routine mail	Triton X-100 buffer	HPLC	HPLC
Jones 2010 [10]*	Yes	-	HR and anti-HbA1c antibody.	TII	TII
Jones 2010 [10]*	Yes	Special shipping	HR and anti-HbA1c antibody.	TII	HPLC
Lacher 2013 [24]	Yes	Packed in dry ice	Extraction buffer	HPLC	HPLC
Lakshmy 2009 [25]	Yes	-	Anti-HbA1c antibody	TII/HPLC	TII
Little 1986 [26]	Yes	-	Water	AC	AC
Lomeo 2008 [27]	-	-	HR	HPLC	HPLC
Tabatabaei-Malazy 2011 [28]*	Yes	-	-	TII	TII
Tabatabaei-Malazy 2011 [28]*	Yes	-	-	TII	TII
Wikblad 1998 [29]	-	Routine mail	Phosphate-citrate buffer	HPLC	TII
**Triglycerides**					
Lakshmy 2010 [30]*	-	Packed in ice	Methanol	Col.	Col.
Lakshmy 2012 [31]*	Yes	Packed in ice	Methanol	Col.	Col.
Quraishi 2006 [32]	Yes	-	Methanol	Col.	Col.
**Total Cholesterol**					
Lacher 2013 [24]	Yes	Packed in dry ice	Deionized water	Col.	Col.
Lakshmy 2010 [30]*	-	Packed in ice	Methanol	Col.	Col.
Lakshmy 2012 [31]*	Yes	Packed in ice	Methanol	Col.	Col.
**HDL**					
Lacher 2013 [24]	Yes	Packed in dry ice	Deionized water	Col.	Col.

^*^Some studies involved multiple samples and are repeated in the table.

The total numbers of participants providing data were 1425 for HbA1_c_, 773 for triglycerides and 1093 for total cholesterol. Study sizes ranged from 30 to 613 participants. The assay methods varied for HbA1_c_ which included immunoturbidimetric, high performance liquid chromatography (HPLC) and affinity chromatography assays but all studies measuring triglycerides, total cholesterol and HDL used colorimetry.

### HbA1c

**Figure 2:**
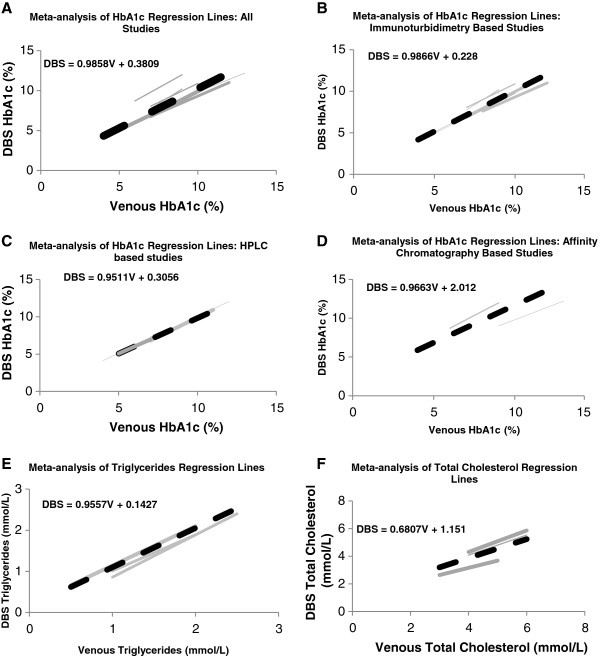
**Individual (solid) and summary (dotted) regression lines showing the associations between results for analyses based upon dried blood spot (DBS) compared to venous (V) samples for haemoglobin A1**_**c **_**(HbA1**_**c**_**) analysed by any method (2A), HbA1**_**c **_**analysed by specific methods (2B to 2D), triglycerides (2E) and total cholesterol (2E). -** Thickness of line increases with sample size. Line length was defined as ±1 standard deviation (SD) of the study (or overall) mean. Where the mean or SD of a study was not available the average for that analysis was used.

For HbA1_c_ the summary regression (DBS = 0.9858 V + 0.3809) (Figure 2) showed close agreement between analyses based upon the venous and DBS sampling methods. There was, however, evidence of heterogeneity between the contributing regression lines for the intercepts (Cochran’s Q-test p < 0.001 and I^2^ = 98%) but not the slopes (p = 0.833 and I^2^ = 0%). Subsidiary analyses by assay method showed that the heterogeneity was partially attributable to different results for the two studies that used affinity chromatography. Funnel plots did not provide clear evidence of publication bias (Additional file 1: Figures S1 and S2).

### Blood lipids

The summary regression line for total cholesterol (DBS = 0.6807Venous + 1.151) (Figure 2) indicates a requirement for moderate adjustment of values based upon analyses of DBS samples to obtain estimates equivalent to standard analyses based upon venous samples. The regression lines for the two studies contributing to this meta-analysis were directly comparable in terms of both slope and intercept although both were derived from studies done at the same investigational centre. For triglycerides, the summary regression for the three contributing studies showed a close association between the results obtained for the two methods (DBS = 0.9557Venous + 0.1427) (Figure 2) without any evidence of heterogeneity between the three results. Only one data set was available for HDL.

### Storage

Data about the circumstances and duration of storage of DBS samples were inconsistently reported with few data to describe whether the analysis findings were affected by extended storage duration or different storage temperatures. From the limited data available it was concluded that DBS samples collected for assay of HbA1_c_ and intended for HPLC analysis can be stored for 5 days at room temperature or for up to 3 years at −70°C [9,11,23]. If analysis by immunoturbidimetry is planned, the data variously suggest that samples can be stored safely at room temperature for up to 44 days [10], at 4°C for up to 15 days [25] and storage at −80°C for up to 3 months [33]. For total cholesterol samples were reported as stable for up to 1 month at room temperature [30,32] and up to 3 months at 4°C [32], and for triglycerides up to 1 month at room temperature and up to 2 months at 4°C [30].

## Discussion and conclusion

These analyses identified clear associations between assay results based upon blood samples collected using traditional venous approaches and blood samples collected using DBS techniques, for both HbA1_c_ and selected blood lipids. The data provide a strong rationale for the further investigation of DBS sample collection techniques although also serve to highlight a number of areas that require further exploration before the method is considered mainstream in this field. If, however, standards and calibrations can be agreed, as has been achieved in other fields of research [23,31,37], the DBS method does appear to have significant potential to address the logistical challenges of venous sampling for studies of metabolic risks in resource poor settings [17].

The differences between the intercepts of the regression lines obtained for the various analytic methods used for assay of HbA1_c_ require careful consideration in terms of their implications. If the variation is due to the analytic method selected then it will be necessary to recommend a standard approach for each analyte of interest. However, while the analytic method is the obvious explanation for the observed variation it is not possible to exclude alternative causes on the basis of the available data. For example, other aspects of the preparation of the DBS samples such as transportation and extraction were not standardised across the different analytic methodologies and might also be a cause of the differences noted.

**Figure 3:**
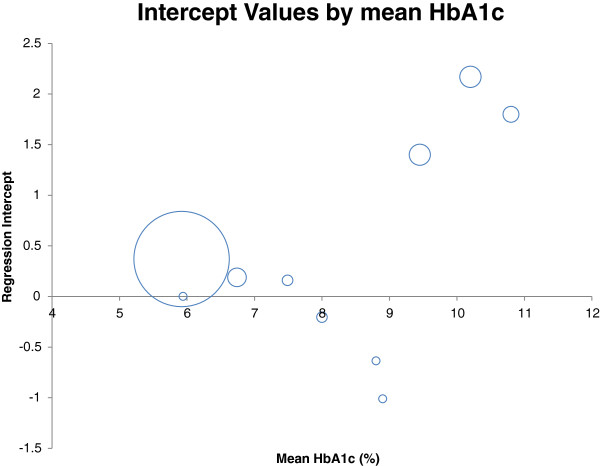
**The mean HbA1c values from the studies are compared with the regression intercept the study generated.** The marker size is proportional to study size.

**Figure 4:**
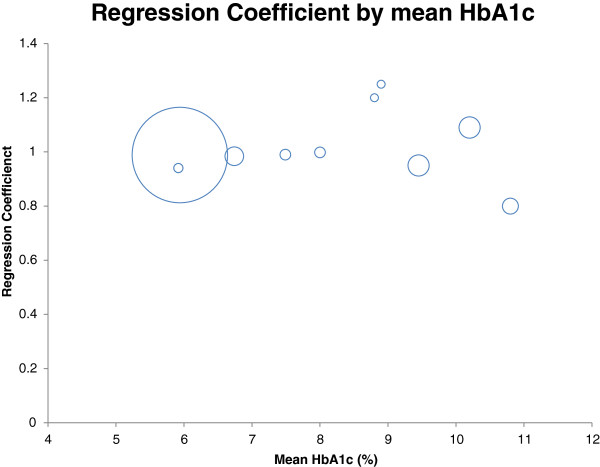
**The mean HbA1c values from the studies are compared with the regression coefficient the study generated.** The marker size is proportional to study size.

The incomplete and summary nature of the data available for analysis placed significant constraints upon the extent to which the results could be explored in this overview. In particular, measures of variance of the data were unavailable for most contributing studies, requiring that weighting be done by sample size alone [18] with consequent limitations upon the methodologies that could be used to present uncertainty intervals around both the individual studies and the summary estimates. For example, we identified a possible relationship between the regression parameters and the mean HbA1_c_ of the contributing studies suggesting that both the intercept and the slope might change when HbA1c rises above 8% (Figures 3 and 4). This implies a non-linear association of venous with DBS sample results that might require a more nuanced explanation than the simple linear regressions provided here [9]. Removing the studies with high average HbA1_c_ levels from the meta-analysis resulted in a regression line approaching parity (DBS = 0.9553 V + 0.2566) and with a reduced heterogeneity for both the slopes (I^2^ = 0%) and the intercepts (I^2^ = 92%). However, whether this simply reflects a chance finding in the data, or a true variation of the association by mean HbA1_c_ level is still uncertain. Likewise, several studies used the same patients for two rounds of analysis [21,28] and there would therefore have been some correlation between the findings for each. This would not be expected to substantially change the parameter estimates obtained but certainly would increase the uncertainty around them. Our inability to create robust uncertainty intervals around our estimates is the primary weakness of this piece of work. Unfortunately the lipid analyses were even less rigorous, with so few studies, confidence in the meta-analysis is limited. There is much potential for further work in this area to generate reliable regression analyses for use in the field.

On a more positive note, the association between results based on venous and DBS samples appeared to be consistent at the levels of HbA1_c_ at which diabetes mellitus is diagnosed (HbA1_c_ > 6.5% [38]). This implies that DBS samples could already be used for determining the presence or absence of diabetes with reasonable certainty, although measures of the extent to which blood glucose is controlled amongst those with diabetes would be less reliable.

Most of the studies reported some information about DBS sample preparation, transport and storage but the data were provided in diverse formats and were substantively incomplete. While it appears likely that DBS samples are stable for adequate periods of time this is an area that requires systematic evaluation and the development of standardised recommendations prior to widespread roll out of the methodology.

The establishment of World Health Organization ‘’25 by 25” target for the prevention of non-communicable diseases [39] has added urgency to the need for data about the metabolic determinants of cardiovascular risk. With more than three quarters of all chronic disease now occurring in developing country settings, the introduction of low cost research techniques that will provide the data required to inform government decision making is a priority [40]. DBS sample collection methods appear to have great potential for the evaluation of cardiometabolic risk factors at the population level [9,13,27] enabling data collection at scale in areas previously unstudied [15]. There remain, however, important advances to be made in defining standard methodologies and adjustments before the DBS sampling method is confirmed as a sound proxy for traditional venepuncture samples for these types of blood analytes.

## Abbreviations

DBS: Dried blood spot; HbA1c: Haemoglobin A1_c_; HIV: Human immunodeficiency virus; HDL: High density lipoprotein; LDL: Low density lipoprotein; HPLC: High performance liquid chromatography.

## Competing interests

There are no conflicts of interest to be declared.

## Authors’ contributions

EA drafted the manuscript and researched data. PD researched data. CC reviewed/edited the manuscript. BN contributed to the study design and re-drafting of the manuscript. Guarantors are EA and BN. All authors’ read and approved the final manuscript.

## Pre-publication history

The pre-publication history for this paper can be accessed here:

http://www.biomedcentral.com/1472-6890/14/21/prepub

## Supplementary Material

Additional file 1: Figure S1 Funnel plot of the HbA1c regression coefficients. **Figure S2.** Funnel plot of the HbA1c regression intercepts. Click here for file
