# Supplementary material for: Comparability of HbA1c and lipids measured with dried blood spot versus venous samples: a systematic review and meta-analysis
Source: BMC Clin Pathol. 2014 May 12;14:21. doi: 10.1186/1472-6890-14-21 (PMC4101836; doi:10.1186/1472-6890-14-21)
Supplement: Additional file 1: Figure S1 — Funnel plot of the HbA1c regression coefficients. Figure S2. Funnel plot of the HbA1c regression intercepts. [file 1472-6890-14-21-S1.pdf]

Additional File

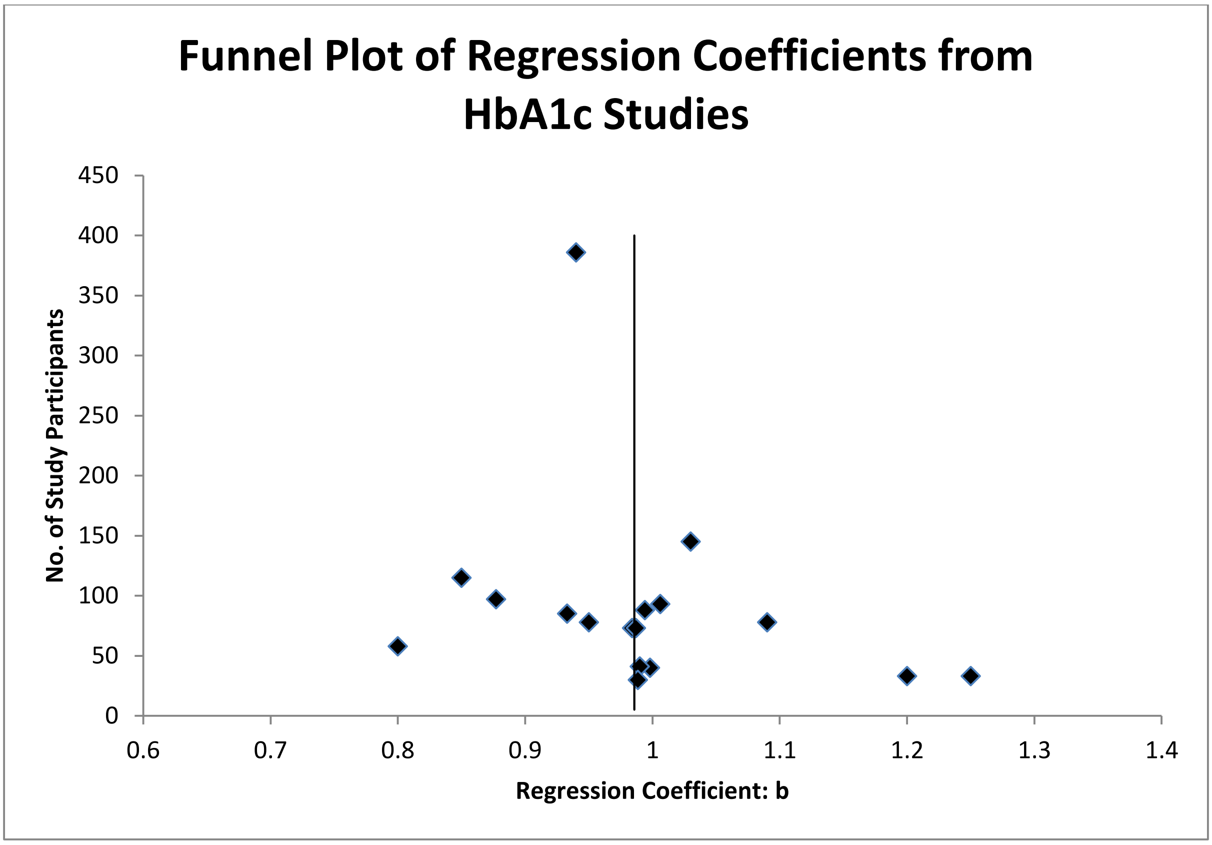

Supplementary Figure 1. Funnel plot of the HbA1c regression coefficients.

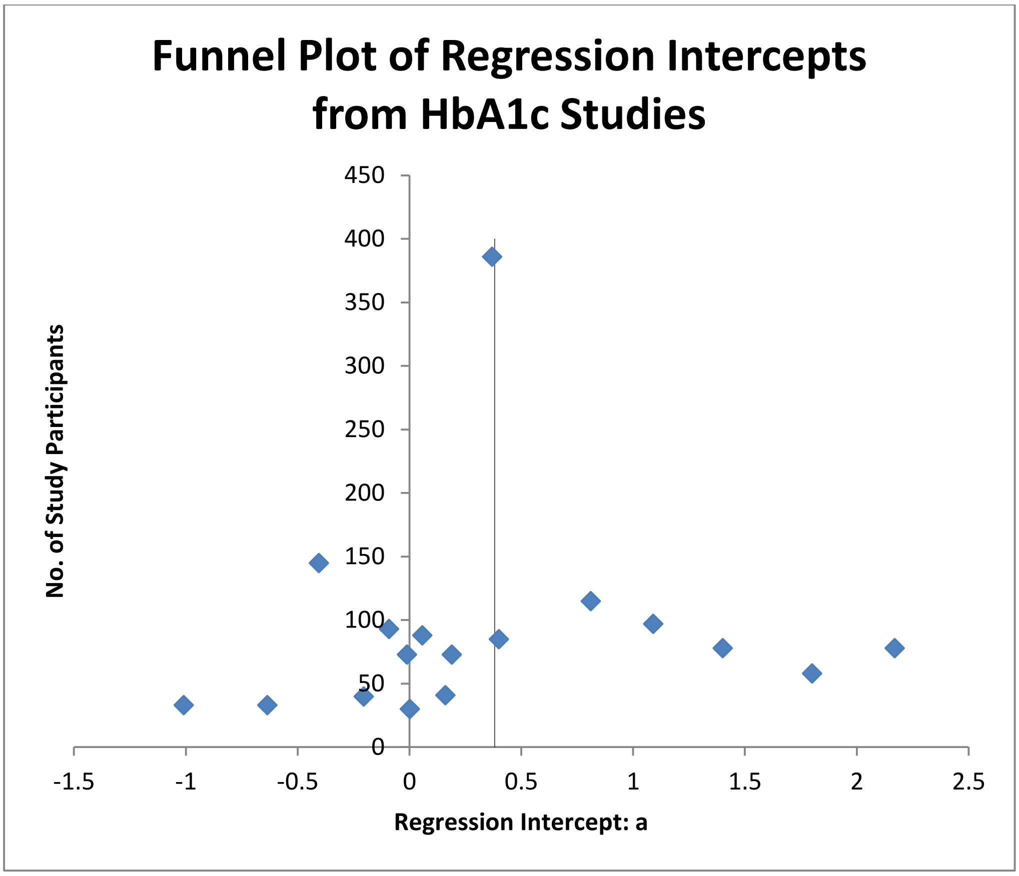

Supplementary Figure 2.Funnel plot of the HbA1c regression intercepts.
